# The effect of UNIMMAP multiple micronutrient supplements versus iron-folic acid and placebo in anemia reduction among women of reproductive age in Kebribeyah Woreda, Somali Regional State, Ethiopia: a study protocol for a community-based individual RCT

**DOI:** 10.1186/s13063-024-08024-w

**Published:** 2024-03-06

**Authors:** Desalegn Kuche, Zeweter Abebe, Masresha Tessema, Meron Girma, Alemayehu Hussen, Kaleab Baye, Barbara J. Stoecker

**Affiliations:** 1 Ethiopian Public Health Institute, Addis Ababa, Ethiopia; 2 Oklahoma State University, Stillwater, OK USA; 3 Addis Ababa University, Addis Ababa, Ethiopia

**Keywords:** UNIMMAP-MMS, IFA, Anemia, Trial, Women of reproductive age

## Abstract

**Background:**

Women of reproductive age (WRA) in developing countries are often at risk of micronutrient deficiencies due to inadequate intakes and excessive losses.

**Objective:**

The purpose of this trial is to assess the effectiveness of United Nations International Multiple Micronutrient Antenatal Preparation-Multiple Micronutrient Supplements (UNIMMAP-MMS) versus iron-folic acid (IFA) among WRA in reducing anemia.

**Methods:**

Three parallel groups of WRA will participate in a community-based, individually randomized, double-blinded, placebo-controlled superiority trial. After consent, the sample of 375 mildly or moderately anemic women based on hemoglobin by Hemocue will be randomly assigned across two interventions and one control arm. Trial participants in intervention arms will receive UNIMMAP-MMS or IFA while those in the control arm will receive placebos twice a week for 17 weeks. The primary outcome will be a change in mean hemoglobin (Hb) concentrations. Outcome assessors and study participants will be blinded to the type of supplements and study arm.

**Discussion:**

The World Health Organization (WHO) added UNIMMAP-MMS to its essential medicine lists in 2021 but recommended rigorous study. Several factors in addition to inadequate intakes of iron and folic acid contribute to the high prevalence of anemia among WRA in the Somali region. The findings of this study will provide evidence on the effect of UNIMMAP-MMS and IFA on Hb concentrations and anemia prevalence among anemic WRA.

**Trial registration:**

ClinicalTrials.gov NCT05682261. Registered on January 12, 2023.

## Administrative information

Note: the numbers in curly brackets in this protocol refer to SPIRIT checklist item numbers. The order of the items has been modified to group similar items (see http://www.equator-network.org/reporting-guidelines/spirit-2013-statement-defining-standard-protocol-items-for-clinical-trials/).

**Table:** 

Title {1}	The effect of UNIMMAP-multiple micronutrient supplements versus iron-folic acid and placebo in anemia reduction among women of reproductive age in Kebribeyah Woreda, Somali Regional State, Ethiopia: A study protocol for a community-based individual RCT.
Trial registration {2a and 2b}	Registered prospectively on January 12, 2023, at ClinicalTrials.gov (ID: NCT05682261)
Protocol version {3}	Version 3: 2024-February 19
Funding {4}	Financial support was obtained from the European Union through the Ethiopian Public Health Institute (EPHI) with grant number 2017/387–643.
Author details {5a}	^1^Ethiopian Public Health Institute, Addis Ababa, Ethiopia ^2^Oklahoma State University, Stillwater, OK, USA ^3^Addis Ababa University, Addis Ababa, Ethiopia
Name and contact information for the trial sponsor {5b}	Ethiopian Public Health Institute, Food Science and Nutrition Research Directorate, 1242, Addis Ababa, Ethiopia info@ephi.gov.et
Role of sponsor {5c}	The sponsoring institute (EPHI) is a federal government organization engaged in conducting and coordinating research activities at the national level in Ethiopia. Sources of funding had no role in determining study protocol and will not be involved in data collection, analysis, or interpretation.

## Introduction

### Background and rationale {6a}

Anemia affects almost one-third of the population of the world and is a major public health problem globally [1, 2]. The 2010 Global Burden of Disease (GBD) analysis revealed a worldwide anemia prevalence of 32.9% [3]. According to the 2020 Global Nutrition Report, women of reproductive (WRA) are particularly affected by anemia with 32.8% of adolescent girls and WRA (18–49 years old) experiencing anemia [4]. Moreover, all countries in the world are “off course” to achieve the target of the World Health Assembly for a 50% reduction in anemia among WRA by 2025 [4].

Anemia is a serious public health problem in Ethiopia. In 2013, the prevalence of anemia among both sexes in Ethiopia was 25.6% [5]. The 2016 Ethiopian Demographic and Health Survey (EDHS) and other national-level studies indicated that anemia prevalence is high among WRA [6–8]. According to the 2016 EDHS, anemia prevalence was 24% nationwide among WRA, with the Somali Regional State having the highest prevalence (59.5%) [8]. In addition, the 2016 Ethiopian National Micronutrient Survey (ENMS) indicated that iron deficiency anemia (IDA) was the highest (25%) in the Somali Regional State. Despite the high prevalence of anemia, the 2016 ENMS revealed that the prevalence of IDA among WRA was low at the national level. The survey found that IDA was 4.7% when assessing anemic women (Hb < 12 g/dL) with low serum ferritin (ferritin ≤ 15 µg/L), whereas IDA was 5.8% when measuring anemic women (Hb < 12 g/dL) with high soluble transferrin receptors (sTfR) (sTfR ≥ 4.4 mg/L) [7]. A comparable prevalence of IDA (5%) was observed among WRA in Sidama Zone, Southern Ethiopia [9].

Several studies have identified different contributors to overall anemia [5, 6, 9, 10]. According to the 2013 GBD analysis, the predominant cause of anemia across the globe was iron deficiency — contributing about 59.2% to anemia in women globally. Additionally, hemoglobinopathies contributed about 11.6%. The other factors that contribute to anemia include gastrointestinal losses (5.3%), gynecologic conditions (5.1%), and malaria (4.9%) [5]. However, estimates of the contribution of iron deficiency to anemia in women vary widely depending on the iron status indicators used. The percentage of anemia caused by iron deficiency among WRA ranges from 25.5% when measuring inflammation-adjusted body iron stores (BIS) as an indicator, 26.8% when using inflammation-adjusted-ferritin, and up to 50% when measuring sTfR [10].

Iron plus folic acid (IFA) and United Nations International Multiple Micronutrient Antenatal Preparation Multiple Micronutrient Supplements (UNIMMAP-MMS) are recommended public health interventions for anemia control [1, 11–13]. The World Health Organization (WHO) recommends a relatively high dose of weekly IFA supplementation for WRA when the prevalence of anemia is greater than 20% [12]. Multiple micronutrient supplements should be promoted to treat co-occurring micronutrient deficiencies in developing nations where micronutrient deficiencies are common [14, 15]. The WHO with its partners suggested pilot studies with weekly or twice weekly UNIMMAP-MMS formulations for non-pregnant women [16]. In 2021, the WHO included the UNIMMAP-MMS in its essential list of medicines and recommended its use with rigorous research for WRA, especially during pregnancy [17]. The multiple micronutrient supplements technical advisory group (MMS-TAG) indicated that UNIMMAP-MMS is efficacious, safe, and cost-effective in controlling micronutrient deficiencies among women [18].

A recent review conducted by Gomes et al. (2022) indicated that MMS containing 30 mg of iron had a comparable effect to IFA with 60 mg of iron on anemia among third-trimester pregnant women. In this review, the investigators noted an evidence gap regarding the effects of MMS with 30 mg of iron on IDA in WRA in a population with a high prevalence of anemia [19]. Therefore, this study will compare the effects of UNIMMAP-MMS versus IFA (both with 30 mg of iron) and placebo on hemoglobin (Hb) concentration, overall anemia, and iron deficiency anemia among WRA with anemia in a setting where there is high anemia prevalence.

### Objectives {7}

The primary objective of this trial is to assess the effects of UNIMMAP-MMS on Hb concentrations in comparison with IFA among WRA. The secondary objective is to assess the effects of these supplements on serum ferritin concentrations and IDA.

### Trial design {8}

The study will be a community-based, individually randomized, double-blind placebo-controlled, superiority (20,21) trial. The trial will have three arms: two intervention arms and one control arm. Women will be randomly assigned to receive UNIMMAP-MMS, IFA supplements, or a placebo (control) twice per week. Given that UNIMMAP-MMS contains multiple vitamins and minerals, we assume it will be superior to IFA because dietary intake of several other vitamins and minerals in addition to iron and folate also may be inadequate for these women.

## Methods: participants, interventions, and outcomes

### Study setting {9}

**Fig. 1:**
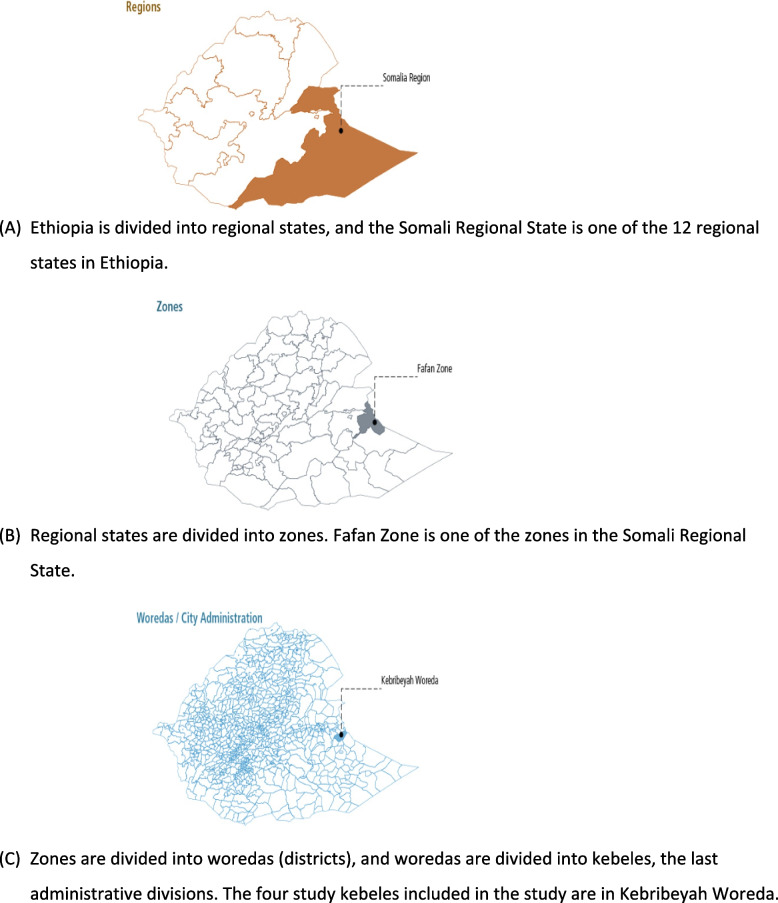
Maps of the study areas illustrating the study region (**A**), zone (**B**), and woreda (**C**). **A** Ethiopia is divided into regional states, and the Somali Regional State is one of the 12 regional states in Ethiopia. **B** Regional states are divided into zones. Fafan Zone is one of the zones in the Somali Regional State. **C** Zones are divided into woredas (districts), and woredas are divided into kebeles, the last administrative divisions. The four study kebeles included in the study are in Kebribeyah Woreda

The study will be conducted in the Somali Regional State among 18–49-year-old non-pregnant WRA. The Somali Regional State is in the east and southeast parts of Ethiopia (see Fig. 1). Figure 1 illustrates the map of the study area. The predominant occupation in the region is pastoralism followed by agropastoralism. The Somali Regional State is divided into 93 woredas made up of 1224 kebeles [8, 20].

**Fig. 2:**
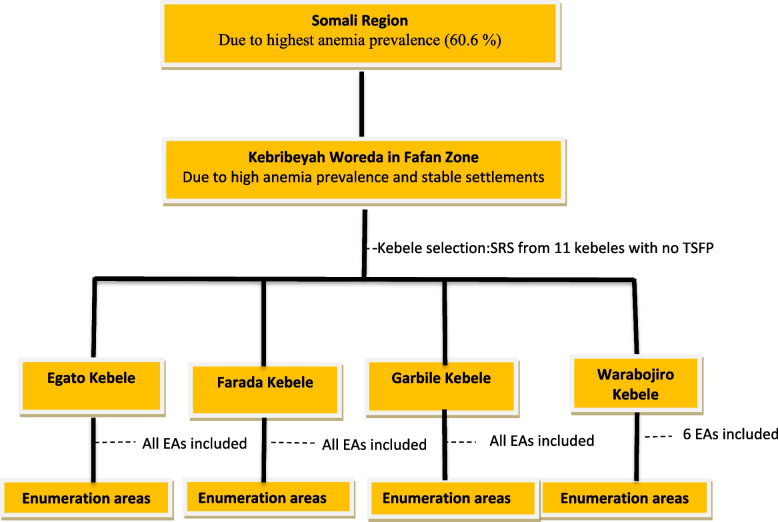
A flow diagram illustrating the study sites and their selection criteria. SRS, simple random sampling; TSFP, Targeted Supplementary Feeding Program, EAs, Enumeration Areas

From the 93 woredas (districts) in the Somali Regional State, Kebribeyah Woreda was selected due to its stable settlements (semi-pastoralist) and high prevalence of anemia among WRA [8]. The average altitude of the woreda is 1530 m above sea level [21]. About 85% of the population lives in rural areas while about 12% are pastoralists [22]. Of the 28 kebeles in Kebribeyah Woreda, 11 kebeles have no Targeted Supplementary Feeding Program (TSFP). From these 11 kebeles, four were selected for the study. Figure 2 displays the study sites and their selection criteria.

### Eligibility criteria {10}

The inclusion criteria are:

1. WRA (18–49 years) with mild or moderate anemia
2. Residence in selected EA for greater than or equal to 6 months
3. Planning to continue living in the study area for the next 6 months

The exclusion criteria are:

1. Age < 18 years or > 49 years
2. Pregnancy
3. Severely ill or incapacitated
4. Severe anemic (Hb < 8 g/dL) or no anemia (Hb > 12 g/dL)

### Who will take informed consent? {26a}

Local health extension workers will be carefully trained and will explain to the eligible study participants about the intervention study. Informed written consent in the local Somali language will be obtained from volunteers in the presence of witnesses from the community before enrollment in the RCT. After consent, the data collectors will conduct an interview, take anthropometric measurements, and collect biological samples (blood, urine, and stool) from the study participants in their homes.

### Additional consent provisions for collection and use of participant data and biological specimens {26b}

Participants will be asked to provide about 15 mL of blood to determine the concentrations of nutrients in their blood and the genetic causes of anemia. We may use the remaining samples for future research purposes such as genetic analysis of zinc deficiency.

## Interventions

### Explanation for the choice of comparators {6b}

This trial will consist of a placebo-only control arm and two intervention arms. The participants in the control group will receive a placebo and will have access to the standard of care. All participants in this trial are non-pregnant WRA who are mildly or moderately anemic. Women may seek health care from the local health post or health center any time that they are ill, but Ethiopia has no standard of care protocol for anemia in WRA unless they become severely anemic. Thus, as per Ethiopia’s protocol, only severely anemic women will be referred to the health facility for treatment. In Ethiopia, mildly and moderately anemic women often get nutrition advice as part of health extension packages for the general population, and all participants, regardless of study arm, will have access to standard care. The study participants in the control group will receive standard of care in the health extension packages. At the end of the intervention as compensation, we will give a 17-week supply (34 tablets) of the UNIMMAP-MMS to study participants who were allocated to the control arm. In addition, we will also encourage this group to the health services at the end of the 17-week intervention.

### Intervention description {11a}

At enrollment, the study participants will be randomly allocated to three study groups: (i) UNIMMAP-MMS tablets (UNIMMAP-MMS arm), (ii) IFA arm, or (iii) a placebo (control arm). All arms will be provided supplements twice per week for 17 weeks. The supplements to be given to the participants are based on the recommendation of the WHO and its partner organizations. The UNIMMAP-MMS tablets contain 15 micronutrients including 30 mg of iron. The doses of vitamins and minerals in the UNIMMAP-MMS tablet are vitamin A as retinol acetate (800 µg RAE), vitamin C as ascorbic acid (70 mg), vitamin D as cholecalciferol (5 µg or 200 International Unit (IU)), vitamin E as alpha-tocopherol succinate (10 mg alpha-TE), vitamin B_1_ as thiamin mononitrate (1.4 mg), vitamin B_2_ as riboflavin (1.4 mg), vitamin B_3_ as nicotinamide (18 mg NE), vitamin B_6_ as pyridoxine hydrochloride (1.9 mg), folic acid (680 µg DFE or 400 µg), vitamin B_12_ as cyanocobalamin (2.6 µg), iron as ferrous fumarate (30 mg), iodine as potassium iodide (150 µg), zinc as zinc oxide (15 mg), selenium as sodium selenite (65 µg), and copper as copper oxide (2 mg). The other intervention is a capsule containing iron and folic acid. The doses of iron and folic acid for the trial are a combination of 30 mg of iron as ferrous sulfate and 400 µg of folic acid as per the recommendation of WHO and its partner organizations [12, 16, 18, 23]. The participants in the control group will receive a placebo containing inactive anhydrous lactose.

We will consult the regional and district health officials to confirm there are no ongoing anemia interventions in the selected woreda and kebeles. We will also exclude kebeles with TSFP to avoid potential contamination. During the intervention, we will continue to assess if there are any anemia interventions in the study sites with a weekly monitoring checklist.

### Criteria for discontinuing or modifying allocated interventions {11b}

The intervention may be modified or discontinued based on reports of adverse effects following the Data and Safety Monitoring Board’s (DSMB) advice. In case of reported side effects and allergic reactions, we will stop the supplementation per DSMB advice. If an accidental overdose that might surpass the upper limit for the micronutrient recommendations occurs, the participants will be immediately referred to the local health center or hospital for medical treatment. However, such a problem is unlikely because study supplements will be kept by the project team and not in the participant’s home. We will report any adverse events to the DSMB, Ethiopian Public Health Institute (EPHI) institutional review board (IRB), and Oklahoma State University (OSU) IRB offices.

### Strategies to improve adherence to interventions {11c}

Health extension workers or community health mobilizers will keep each woman’s bottle of allocated supplements and bring them to the home. These workers will supervise the study participants to ensure they take the supplements. During this visit, the health extension workers or community health mobilizers will inform participants of the benefits of micronutrient supplements for anemia reduction which may reduce the attrition rate. In addition, the supplements that study participants are assigned to take will be routinely inspected by the principal investigator (PI) and the supervisor.

### Relevant concomitant care permitted or prohibited during the trial {11d}

Usual care will be available during the trial. The use of prescription medications from medical professionals is permitted. However, MMS or IFA supplements from other sources are prohibited for study participants. If anyone of the participants takes other nutritional supplements, they will be excluded from the study. Furthermore, we will also exclude kebeles (communities) that have TSFP. We will provide UNIMMAP-MMS tablets to severely anemic women who will be excluded from our study and followed as an additional group for the intervention period. Any severely anemic woman will also be referred to the nearby health center or hospital for additional medical care.

### Provisions for post-trial care {30}

At the end of the follow-up period, the control group will receive UNIMMAP-MMS tablets. Additionally, we will connect the study participants with the local health facilities for additional medical care as needed.

### Outcomes {12}

The primary outcome will be a change in Hb concentrations. We will determine hemoglobin concentrations in venous blood samples using HemoCue 301 and report changes in mean Hb concentration between the control and intervention arms compared to the baseline Hb concentration after the 17 weeks of supplementation. Hb concentrations will be adjusted for altitude and smoking status [24]. The secondary outcomes include a change in serum ferritin levels and measurements of nutritional deficiencies including folate, vitamin B_12_, vitamin A, and zinc from serum samples. Serum micronutrient biomarkers will be adjusted for inflammation and infection using C-reactive protein (CRP) and alpha 1-acid glycoprotein (AGP) [25].

### Participant timeline {13}

**Fig. 3:**
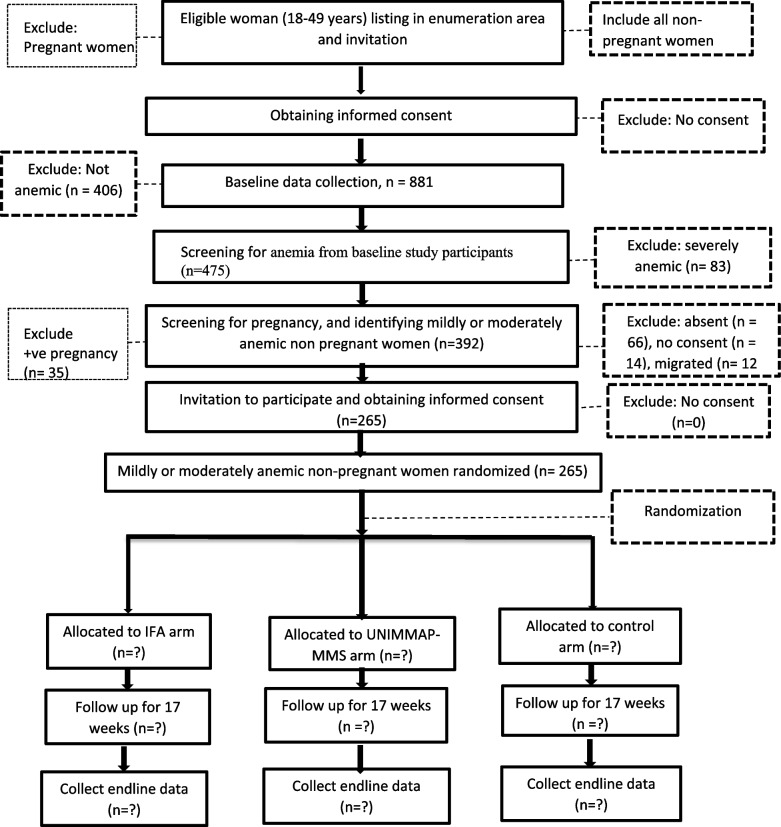
A flow diagram illustrating the recruitment and allocation of participants in the study. *n*, number of study participants planned; IFA, iron-folic acid; UNIMMAP-MMS, United Nations International Multiple Micronutrient Antenatal Preparation-Multiple Micronutrient Supplements

**Fig. 4:**
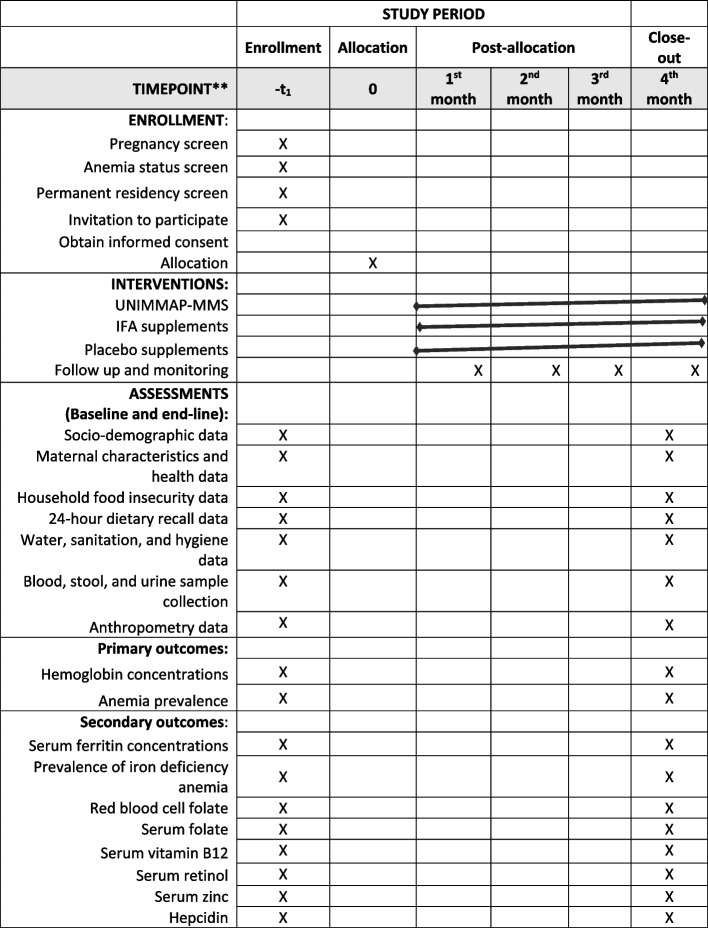
Schedule of enrollment, interventions, and assessments

To identify anemic women and collect baseline data, supervisors and data collectors will do a census to list all eligible WRA in the selected EAs. One woman from each household will be invited to participate in the baseline study if the screening questions and self-report indicate she is not pregnant. After an interview, anthropometric measurements will be taken, and a venous blood sample collected to measure Hb concentration and iron biomarkers. Urine and stool samples will be collected to investigate other potential contributors to anemia. The proposed intervention will begin within four weeks of the completion of the baseline data collection (see Figs. 3 and 4). Figure 3 illustrates the plan for identification and recruitment of study participants and Fig. 4 illustrates the schedule of enrollment, interventions, and assessments.

Women will qualify for the intervention trial if they are mildly (Hb = 11.0–11.9 g/dL) or moderately anemic (Hb = 8.0–10.9 g/dL) at the baseline and if they have a negative pregnancy test. Health extension workers or community health mobilizers will visit each eligible woman in her home to confirm her participation in the intervention study. Written informed consent will be obtained from all women participating in the intervention study. The study participants will then be randomly allocated to the study arms.

When the intervention is completed in 17 weeks, the data collectors again will obtain written informed consent from the participants. Subsequently, they will conduct a final interview, take anthropometric measurements, and collect a venous blood sample again to test Hb concentration and iron status as well as stool and urine samples.

### Sample size {14}

This study is based on repeated measures at the beginning and end of the interventions. Hence, we used G*power analysis for sample size calculations to assess between factors in repeated measures of Analysis of Variance (ANOVA) with 0.25 g/dL (conventional medium effect size for change in Hb concentrations), α error probability = 0.05, power (1-β error probability) = 0.9, number of groups = 3, number of measurements = 2, and correlation among repeated measures = 0.5. A sample size of 156 is determined for the intervention study based on the above information. Including a design effect of 1.5 and a default rate of 20%, the final total sample size is 375. Therefore, about 375 women will be sampled from the four kebeles for the intervention study. We will distribute our sample to the four kebeles based on probability proportional to size (PPS).

### Recruitment {15}

WRA residing in the study areas will be identified through a census for baseline assessment. Health extension workers or community health mobilizers then will visit each mildly or moderately anemic woman in her home and inform her that she is eligible for the intervention study based on her anemia status. Written informed consent will be obtained from all women volunteering for the intervention study. If a woman does not consent or has a positive pregnancy test, she will not be included in the intervention study.

## Assignment of interventions: allocation

### Sequence generation {16a}

All consenting and non-pregnant study participants will be randomly assigned to the three study arms. The allocation sequence for random assignment will be generated using STATA 16.1 (StataCorp LLC, TX, USA).

### Concealment mechanism {16b}

A sequence and a letter representing a study arm will be placed in sealed opaque envelopes by a researcher who is not affiliated with the study. When a woman meets the screening criteria and gives informed consent, she will be assigned an envelope with a letter code corresponding to a study group.

### Implementation {16c}

The allocation sequence will be generated at the coordination center by a non-participating staff of the EPHI and will be maintained confidentially until the completion of the trial. Assignment to one of the intervention groups will be considered as official enrollment in the study group.

## Assignment of interventions: blinding

### Who will be blinded {17a}

In the current trial, the outcome assessors and trial participants will both be blinded. The trial participants and outcome assessor will be blinded to the type of supplements being administered and to the group assignment.

### Procedure for unblinding if needed {17b}

The DSMB will assess any safety concerns if the safety of the supplements is questioned and may advise continuing blinding or breaking codes. We shall proceed as per the DSMB recommendations.

## Data collection and management

### Plans for assessment and collection of outcomes {18a}

Data collectors will use a tablet-based pre-tested questionnaire, which will be administered to participants during an individual face-to-face interview. Data collection will be supervised by experienced supervisors, coordinators, and the PI.

Trained medical laboratory technicians will draw blood samples from participants which will reduce the risk of harm. The medical laboratory technicians will take care of any issues related to blood collection and allergic reactions. In case of emergency, we can take a participant to a nearby health facility with a fieldwork car.

We will assess Hb concentrations and malaria from a whole blood sample (venous) in the field. Also, a complete blood count will be done from the whole blood sample in a medical laboratory to confirm Hb concentrations. In addition, nutritional status of the participants will be assessed which will include serum biomarkers such as hepcidin, ferritin, transferrin receptors, and concentrations of various micronutrients that may impact the primary outcome; these serum biomarkers of micronutrient status will be adjusted for infection and inflammation using the equation developed by Biomarkers Reflecting Inflammation and Nutritional Determinants of Anemia (BRINDA) Working Group [25]. Anthropometric measurements such as body mass index (BMI) and mid-upper arm circumference (MUAC) will further support nutritional assessment of participants.

Urine samples will be collected to test for hematuria due to urogenital schistosomiasis. The respondents will be given a pre-labeled 60 mL urine cup by staff to collect a sample. The urine will be stored in a cold box, transferred to a portable freezer, and subsequently stored at – 20 °C.

Stool samples will be collected to test for soil-transmitted helminths. Study participants will be given a stool collection cup with a pre-attached spatula. A laboratory technician will collect the stool samples from the home as quickly as feasible, add 10% formalin to preserve the parasites, and maintain samples in a cold chain. The stool sample will be tested for total egg counts for helminth infections including hookworms and schistosomiasis [26, 27]. All the data mentioned above will be collected at baseline and repeated for all groups immediately after the intervention is completed.

### Plans to promote participant retention and complete follow-up {18b}

Health extension workers and community health mobilizers who live in the study areas will visit each study participant twice a week on pre-arranged days to deliver and supervise supplements. We will conduct an intention-to-treat analysis by taking into account all participants who will be assigned to study arms including those lost to follow-up.

### Data management {19}

Data will be collected via the ODK tool with Android mobile phone devices. Data will be transferred from the field directly to the central server and stored on this secure server in the information technology (IT) unit at EPHI. We will use unique identifications (numbers, not names) during data analysis. Data will be exported to STATA and/or SAS for cleaning and analysis. Maternal and household characteristics and biochemical parameters will be compiled. Frequency and percentage for categorical variables, mean ± SD for normally distributed continuous variables, and median with interquartile range (IQR) for non-normally distributed continuous variables will be calculated. Normality will be tested using the normal probability plot (quantile–quantile plot) and test statistics.

### Confidentiality {27}

Confidentiality and data safety measures will be employed to prevent violation of privacy. Signed consent forms will be locked in the office of the PI. The data will be kept in a secure server in the IT unit of EPHI. We will have backup data for the interview responses and the laboratory test results in password-protected computers handled by the PI. The researchers in the study team will have access to survey responses and biological sample test results for research purposes. No other persons will have access to the data unless authorized. Stored samples will be identified with barcodes. We will remove names and use unique identifiers (numbers) of participants during data analysis. The master key will be stored in a locked file cabinet in the office of the PI separated from other materials. No personal information will be included during reporting and publication. The master key for personal information will be burned or shredded after publishing key findings in academic journals.

### Plans for collection, laboratory evaluation, and storage of biological specimens for genetic or molecular analysis in this trial/future use {33}

Specimen processing and storage sites (temporary field labs) will be prepared in each kebele to immediately manage the samples coming from the household. All blood samples will be processed within 1 h of collection. Finally, all specimens will be transported to the EPHI to be stored as appropriate until final analyses. Hemoglobinopathies including thalassemia and sickle cell disease will be tested from the whole blood sample for genetic contributors to anemia. The remaining blood samples will be stored for future genetic analysis of zinc deficiency.

## Statistical methods

### Statistical methods for primary and secondary outcomes {20a}

The effects of the intervention on Hb concentrations and serum ferritin levels will be examined with a linear mixed model (LMM) approach which provides flexibility that accommodates both fixed and random effects. Additionally, a generalized linear mixed model (GLMM) will be used to further evaluate the effect of intervention on Hb concentrations and serum ferritin levels. The study will also use Generalized Estimating Equations (GEE) to estimate the population-averaged effects of the intervention on Hb concentrations, serum ferritin levels, iron deficiency anemia, and overall anemia.

Intention-to-treat (ITT) analysis will be a primary analysis approach to evaluate the effect of the interventions on Hb concentrations. Per-protocol (PP) analysis will be used as a secondary analysis approach for the trial which allows determination of the effect of the intervention in a setting with high adherence and low loss to follow-up. The study groups are expected to be comparable because all three study arms will be present in each of the four study kebeles with very similar settings. In addition, participants will be assigned to the study arms at random. The study will provide a comprehensive understanding of the effectiveness of intervention by combining ITT and PP and using LMM, GLMM, and GEE.

### Interim analyses {21b}

The DSMB members may use the results from an interim analysis to suggest continuing or stopping the intervention during the implementation. We may use the capillary blood to test Hb concentrations if the interim analysis is required by the DSMB for a decision.

### Methods for additional analyses (e.g., subgroup analyses) {20b}

We plan to conduct a subgroup analysis on the severity of anemia (mild or moderate) and causes of anemia.

### Methods in analysis to handle protocol non-adherence and any statistical methods to handle missing data {20c}

ITT analysis (all participants as randomized) will be our main analysis strategy. We also will establish criteria and carry out PP analysis. We anticipate high adherence and low loss to follow-up because we are visiting the participants in their homes and giving them the supplements with the help of the hired community health mobilizers and/or health extension workers. To deal with possible missing data, imputations will be considered. Sensitivity analysis will be performed to verify the robustness of the trial results by taking into account different scenarios of managing missing data [28].

### Plans to give access to the full protocol, participant-level data and statistical code {31c}

Upon publication, the protocol will be accessible online. Whenever needed, we are willing to make our statistical codes available to interested parties upon request after publication. The individual-level data sharing will be governed by the data-sharing policy of the EPHI. However, interested researchers may seek access to the data by submitting a data request form. Upon approval, the data set may be shared.

## Oversight and monitoring

### Composition of the coordinating center and trial steering committee {5d}

The EPHI will be the coordinating center for this trial. The trial will be coordinated and overseen by advisory committee members established from EPHI and Addis Ababa University (AAU) in Ethiopia and OSU in the USA. The composition of the committee includes nutritionists, statisticians, and medical laboratory professionals. The PI will periodically provide the advisory committee with an update on the progress of the trial. When necessary, the advisory group will schedule meetings to assess the trial’s progress.

### Composition of the data monitoring committee, its role, and reporting structure {21a}

The progress of this trial will be monitored by an independent DSMB. We will have a DSMB at EPHI consisting of a medical doctor, a statistician, a representative of an institutional review board, and a nutritionist to monitor the safety of supplementation during the intervention. The board will have its charter and checklists including a case report form (CRF).

We will report any event that may happen concerning the trial to the EPHI and OSU IRB offices and to the DSMB during the intervention. The DSMB board members will access all registry information, checklists, and monitoring forms to assess and analyze data for any adverse events. In addition, the board members may request an interim analysis result to suggest actions for the ongoing trials.

### Adverse event reporting and harms {22}

We anticipate no adverse effect on participants related to the UNIMMAP-MMS or IFA supplementation. These supplements are available on the market and recommended by WHO for micronutrient deficiency prevention. The subjects will be referred to the nearest health center or hospital to get medical care if any unexpected problem occurs. In case of reported side effects and allergic reactions, we will strictly monitor the participant to stop the supplementation. We will report any adverse events to the DSMB, and the EPHI and OSU IRB offices.

### Frequency and plans for auditing trial conduct {23}

An independent monitoring team may visit all study sites and study arms on their schedules if there are any reported adverse events or to investigate high withdrawals in the study arms. The investigators will monitor the trial according to their implementation plan. In addition, the advisory committee members will monitor the progress of the trial. However, the commercial sponsors will have no role in monitoring the trial.

### Plans for communicating important protocol amendments to relevant parties (e.g. trial participants, ethical committees) {25}

If an amendment is necessary and appropriate, we will amend the protocol and communicate it to both the EPHI and OSU IRB offices. We will announce and request an amendment to IRB offices in a formal email to the offices or by filling out an amendment form from the IRB offices. Any amendments will need to be approved by the IRB offices and the DSMB before including the amendments in the protocol. When approved, the protocol will be updated to include the modifications, and all trial modifications will be documented.

### Dissemination plans {31a}

This trial will generate data on the mean Hb concentrations and on reductions in iron deficiency anemia and overall anemia following a four-month micronutrient intervention. The key findings from this trial will be shared with the Federal Ministry of Health (FMOH) of Ethiopia, the Somali Regional State Health Bureau, and Kebribeyah Woreda Health Office. The findings will contribute to addressing anemia and iron deficiency among women, particularly in the rural kebeles of the Kebribeyah woreda in the Somali Regional State. Additionally, the academic community and nutrition stakeholders will be informed of the findings from the trial. The findings will also be presented at professional conferences and published in peer-reviewed journals.

## Discussion

The extent of iron deficiency anemia has been debated in Ethiopia. The serum ferritin test results from the nationally representative micronutrient survey indicated that 10% of WRA were iron deficient [7]. In addition, Andersen et.al in their anemia etiology study showed that iron deficiency measured as low serum ferritin was 13.8% among WRA in Ethiopia [6]. As indicated in both surveys, iron deficiency and iron deficiency anemia were not very widespread among WRA in Ethiopia [6, 7]. On the other hand, the prevalence of anemia is high among WRA, especially in the Somali Regional State [8].

According to national dietary surveys, the majority of the population in Ethiopia, including women, receive an adequate amount of iron. The median iron intake was nearly adequate for WRA at the national level in Ethiopia even though there is a large regional variation with the highest inadequate iron intake (84.3%) in the Somali Regional State [6, 29]. Based on Ethiopia’s 2013 National Food Consumption Survey (NFCS), the median iron intake nationally among WRA was 43.7 mg/day which is considerably above the recommended dietary allowance (RDA). However, the median intake of iron for WRA in the Somali region was 14.5 mg/day. Food insecurity prevalence is high in Ethiopia [30]. The national mean ± standard deviation (SD) energy intake for WRA was 1726 ± 768 kcal whereas the mean energy intake for the WRA in the Somali Regional State was 1293 ± 668 kcal which was the lowest mean energy intake compared to other regions. According to the 2013 NFCS, carbohydrates contributed 76.2% of the mean energy intake, protein contributed 8.6% of energy, and fat contributed 15.7% of the mean energy intake for WRA in the Somali Regional State [29].

The cereal and grain food group made up about 67.9% of women’s diets in the Somali region per the 2013 NFCS, while animal-source foods, excluding dairy products, contributed only about 1.5% of the diet. However, dairy foods (milk, cheese, and whey) contributed 13.9% to the food consumption practices of women’s diet. Moreover, vitamin A-rich fruit and vegetable consumption in women’s diets was very small across all regions in Ethiopia, ranging from 0.2% in the Somali region to 10.4% in the Gambella region. Additionally, a relatively high phytate-to-iron molar ratio (3.16) reported in the 2013 NFCS may lead to a potential reduction in iron bioavailability [29].

A WHO report and some studies indicated that about 50% of anemia is caused by iron deficiency [5, 31]. Other studies indicated that a variety of different factors beyond iron deficiency are likely to be contributors to the high prevalence of anemia [6, 10]. Andersen and colleagues found that low serum ferritin and folate, malaria, and high CRP were risk factors for anemia among WRA in Ethiopia [6]. Anemia is caused, among other factors, by the adverse effects of micronutrient deficiencies, particularly those of iron, folate, vitamin A, and vitamin B_12_ [2, 32]. In addition, the magnitude of anemia is affected in the presence of inflammation [6, 10].

Health of women should be an important consideration prior to conception in order to improve individual health and pregnancy outcomes [33]. When an anemic woman becomes pregnant, anemia not only puts her own health at risk by sometimes causing fatigue, loss of productivity, breathlessness, dizziness, or headache but also can negatively impact the unborn child by increasing the likelihood of low birth weight, preterm delivery, stillbirth, low iron stores at birth or low Hb concentrations [34, 35].

A review of studies indicates that iron treatment, in any form, may enhance Hb and ferritin concentrations to reduce the burden of anemia [36]. This trial will generate evidence regarding how UNIMMAP-MMS and IFA affect iron deficiency and overall anemia. Studies in other contexts have demonstrated that using multiple micronutrients to control anemia is more cost-effective than using very few micronutrients or a single micronutrient [18].

## Trial status

We expect to start participant recruitment in the fourth week of March 2023. We anticipate that the recruitment of the participants for the intervention trial will be completed by the end of March 2023 and that the intervention will be started soon after.

## Data Availability

All investigators will have access to the trial dataset. Ethiopian Public Health Institute shall be the owner of the dataset. According to the data sharing policy, the dataset may be shared upon official request to EPHI.

## Abbreviations

AAU: Addis Ababa University
AGP: Alpha-1-acid glycoprotein
alpha-TE: Alpha-tocopherol equivalent
ANOVA: Analysis of variance
BIS: Body iron stores
BMI: Body mass index
BRINDA: Biomarkers Reflecting Inflammation and Nutritional Determinants of Anemia
CRF: Case report form
CRP: C-reactive protein
DFE: Dietary folate equivalent
DSMB: Data and Safety Monitoring Board
EA: Enumeration area
EDHS: Ethiopian Demographic and Health Survey
ENMS: Ethiopian National Micronutrient Survey
EPHI: Ethiopian Public Health Institute
FMOH: Federal Ministry of Health
GBD: Global Burden of Disease
GEE: Generalized Estimating Equations
GLMM: Generalized linear mixed models
Hb: Hemoglobin
IDA: Iron deficiency anemia
IFA: Iron-folic acid
IRB: Institutional Review Board
IT: Information technology
ITT: Intention-to-treat
IU: International Unit
LMM: Linear mixed model
MMS-TAG: Multiple Micronutrient Supplements Technical Advisory Group
MUAC: Middle-upper arm circumference
NE: Niacin equivalent
NFCS: National Food Consumption Survey
ODK: Open Data Kit
OSU: Oklahoma State University
PI: Principal investigator
PLC: Private Limited Company
PP: Per protocol
PPS: Probability proportional to size
RAE: Retinol activity equivalent
RCT: Randomized controlled trial
SD: Standard deviation
SRS: Simple random sampling
sTfR: Soluble transferrin receptor
TSFP: Targeted Supplementary Feeding Program
UNIMMAP-MMS: United Nations International Multiple Micronutrient Antenatal Preparation- Multiple Micronutrient Supplement
WHO: World Health Organization
WRA: Women of reproductive age
